# Ambivalent Outcomes of Cell Apoptosis: A Barrier or Blessing in Malaria Progression

**DOI:** 10.3389/fmicb.2016.00302

**Published:** 2016-03-15

**Authors:** Parik Kakani, Sneha Suman, Lalita Gupta, Sanjeev Kumar

**Affiliations:** Molecular Parasitology and Vector Biology Lab, Department of Biological Sciences, Birla Institute of Technology and SciencePilani, India

**Keywords:** cell apoptosis, *Plasmodium*, mosquito, midgut epithelium, hepatocytes, erythrocytes

## Abstract

The life cycle of *Plasmodium* in two evolutionary distant hosts, mosquito, and human, is a complex process. It is regulated at various stages of developments by a number of diverged mechanisms that ultimately determine the outcome of the disease. During the development processes, *Plasmodium* invades a variety of cells in two hosts. The invaded cells tend to undergo apoptosis and are subsequently removed from the system. This process also eliminates numerous parasites along with these apoptotic cells as a part of innate defense against the invaders. *Plasmodium* should escape the invaded cell before it undergoes apoptosis or it should manipulate host cell apoptosis for its survival. Interestingly, both these phenomena are evident in *Plasmodium* at different stages of development. In addition, the parasite also exhibits altruistic behavior and triggers its own killing for the selection of the best ‘fit’ progeny, removal of the ‘unfit’ parasites to conserve the nutrients and to support the host survival. Thus, the outcomes of cell apoptosis are ambivalent, favorable as well as unfavorable during malaria progression. Here we discuss that the manipulation of host cell apoptosis might be helpful in the regulation of *Plasmodium* development and will open new frontiers in the field of malaria research.

## Introduction

Arthropods are infamous vectors for numerous human diseases that are major public health hazards throughout the world. The causative agents of these diseases include helminths, protozoa, bacteria, and viruses. Among the large number of vector-borne infections, malaria is included in the list of top five infectious diseases. It is caused by an apicomplexan parasite *Plasmodium* and transmitted by the *Anopheles* mosquito among humans. This disease is reported in over 90 countries primarily in tropical and subtropical regions in sub-Saharan Africa, Central and South America, the Caribbean island of Hispaniola, the Middle East, the Indian subcontinent, South-East Asia, and Oceania. On an average more than 198 million people are infected and 584,000 people die due to malaria every year worldwide (World Health Organization [WHO], 2014).

**FIGURE 1:**
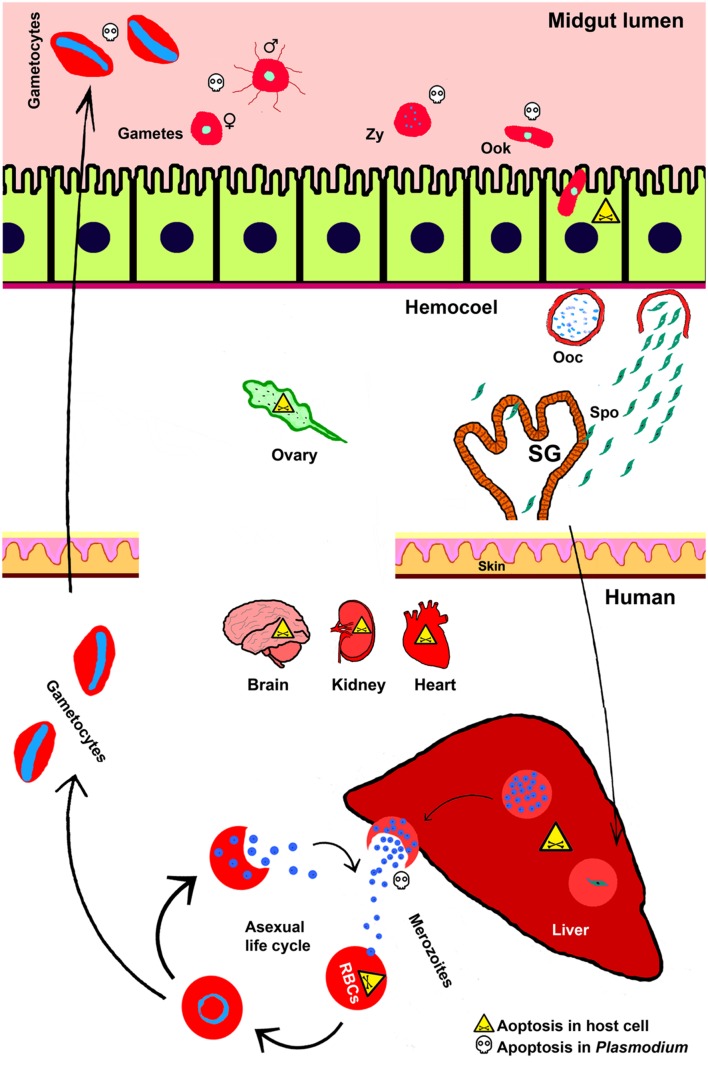
**Apoptosis during *Plasmodium* life cycle in invertebrate and vertebrate hosts.** Life cycle of *Plasmodium* is completed in two hosts. The sexual life cycle occurs in mosquito and asexual life cycle takes place in the vertebrate host such as, human. Various stages of *Plasmodium* development and the phenomena of cell death in both the hosts and parasites are depicted here. Zy, Zygote; Ook, ookinetes; Ooc, oocysts; Spo, sporozoites; SG, salivary gland.

The malaria parasite *Plasmodium* completes its life cycle in two evolutionary distant hosts; mosquito and a vertebrate such as human. The sexual life cycle is completed in female *Anopheles* mosquito (a definitive primary host) and asexual life cycle predominates in human (a secondary host). The mosquito ingests *Plasmodium* gametocytes stages that undergo fertilization and form zygote inside the midgut lumen. Further, in the same compartment, the formation of ookinetes from zygote takes place near about 15h after ingestion. Ookinete traverses the midgut epithelium around 24h of ingestion and develops into an oocyst in the space between midgut epithelium and basal lamina (**Figure 1**). Oocyst matures approximately after 10 days and then releases 1000s of sporozoites into the mosquito haemocoel. Sporozoites circulate throughout the hemolymph and some of them end up in salivary glands. Sporozoites entered the salivary gland further undergo maturation and are ready to be injected into the new human host during the subsequent feeding (Zheng, 1997; Beier, 1998; Sinden, 2002).

*Plasmodium* cycle in human host begins with the entry of mosquito-injected sporozoites into the circulating blood. These sporozoites migrate toward the liver and initiate pre-erythrocytic cycle in hepatocytes that continue approximately for 6–15 days. During this phase, *Plasmodium* undergoes extensive growth and division and at the end, 1000s of merozoites are released into the blood. These merozoites further invade red blood cells (RBCs) and initiate erythrocytic cycle (Frevert, 2004). This asexual erythrocytic cycle further produces more merozoites and at 48 or 72 h, depending on the *Plasmodium* species, they are released from the RBC and immediately invade new erythrocytes. In continuation of the erythrocytic cycle some merozoites differentiate into gametocytes and after ingestion by another female mosquito they continue the sexual cycle as discussed above (Cowman et al., 2012).

It is noteworthy to mention that in both the hosts, *Plasmodium* development takes place in compartments-specific manner and during this process parasite interacts with diverse cell types. During these interactions, apoptosis takes place in the parasites as well as the host cells. Apoptosis is a genetically orchestrated type of cell death that involves numerous distinguishable morphological and cellular alterations (Kerr et al., 1972; Cowman et al., 2012). Generally, the advanced stages of apoptosis are associated with the removal of dying cell from the body system and its replacement by a new cell. Studies revealed that pathogens are eliminated along with the dying cell in case of several diseases, including malaria (Williams, 1994; Sinai et al., 2004).

Cell apoptosis plays an important role in establishment of host–pathogen relationships. The present review identifies the sites of apoptosis in malaria parasite and host cells during *Plasmodium* life cycle. It also highlights the effect of apoptosis on the parasite or host as a strategy by which the parasites proliferate in a healthy environment or a self-defense/repair mechanism by the host. Here, apoptosis of host cells, *Plasmodium* and the outcome of this phenomenon in disease progression has been discussed. Apoptosis of immune cells in both the hosts is not considered as part of this review.

## Apoptosis in *Plasmodium* During Sexual Stages of Development

*Plasmodium* encounters a harsh environment inside the mosquito gut that creates somewhat a bottleneck situation to the parasite numbers at different stages of development such as, gamete, zygote, and ookinete stages (Chose et al., 2003). Majority of gametocytes that arrive in the midgut lumen fail to develop further and on an average approximately 80% of them undergo apoptosis (Sinden, 1999; Sinden and Billingsley, 2001). Studies analyzed the mode of cell death in *Plasmodium berghei* (mouse malaria) sexual stages found that zygotes and ookinetes exhibit the characteristic apoptotic cell morphology revealed by DNA fragmentation, chromation condensation and phosphatidylserine (PS) translocation (Al-Olayan et al., 2002).

The apoptosis in *Plasmodium* may be a natural way to select the most potent or best ‘fit’ parasite that can carry forward the malaria cycle in mosquito where it may also reduce the parasite burden for better survival of the insect host. This indicates that an increased load of *Plasmodium* infection might have deleterious effects in the mosquito host. Similar parasitic behavior is also reported in *Leishmania* where promostigote stage reveals apoptotic features during their development inside the sand fly gut. Apoptotic parasites die and show philanthropic behavior toward the viable parasites. This phenomenon maintains a vital association for the survival of both parasites and sand fly (Shaha, 2006; Van Zandbergen et al., 2006).

It is also possible that mosquito internal environment factors such as, immune components of the ingested blood, the natural gut symbionts or mosquito innate immunity might be regulating the process of apoptosis in *Plasmodium*. The immune components in mosquito ingested blood include white blood cells (WBCs), complement system proteins, cytokines and reactive oxygen or nitrogen species (Lensen et al., 1998; Ramiro et al., 2011; Simon et al., 2013). Previous reports found that WBCs have phagocytic activity against *P. falciparum* and *P. berghei* gametocytes/gametes in *Anopheles gambiae* midgut. The removal of WBCs form the ingested blood significantly reduced the number of apoptotic ookinetes. In addition, the nitric oxides (NO) generated by activated WBCs also induce parasite death (Lensen et al., 1997; Muniz-Junqueira et al., 2001). Cytokines such as, TNF-α and TGF-β1 present in the ingested blood meal also have anti-plasmodial effects. TNF-α inhibits the male gamete formation (called exflagellation) in *P. berghei* that is mediated through leucocytes generated reactive nitrogen species (RNS; Naotunne et al., 1993; Ali et al., 2010; Ramiro et al., 2011). TGF-β1 in fact induces *A. stephensi* nitric oxide synthase (NOS) at low concentrations and suppresses the parasite numbers (Luckhart et al., 2003). The human complement system remains active up to 6 h in the mosquito midgut. A direct effect of the complement system in preventing gametogenesis has been observed in case of *P. falciparum* development. However, oocysts and zygotes have learned to nullify the complement-mediated lysis through surface binding of factor H that inactivates complement effector protein C3b. This seems to be a co-evolved mechanism of protection in these complement-sensitive stages (Simon et al., 2013). The role of mosquito gut flora in the regulation of *Plasmodium* development is reported by several laboratories. In a simple way, the reduction of microbial communities in antibiotics fed *Anopheles* mosquitoes increases their susceptibility to malaria parasite infection (Boissière et al., 2012; Minard et al., 2013; Kajla et al., 2015a). These observations are important in terms of manipulating the endogenous microbial flora of the mosquito to control their vectorial capacity and this area is under investigation by many researchers.

Gene silencing studies revealed that a number of mosquito immune genes regulate parasite load. For example, clip-domain serine protease (CLIPC2) plays an anti-plasmodial role (Blumberg et al., 2013). Similarly, thioester containing protein 1 (TEP1), an opsonin, induces parasite melanization and inhibits *Plasmodium* development (Blandin et al., 2004; Blumberg et al., 2013). In addition, mosquito generated NO and reactive oxygen species (ROS) have been reported to trigger apoptosis in *Plasmodium* ookinetes. Studies demonstrated that *in vitro* exposure of *Plasmodium* ookinetes with sodium nitroprusside, the NO producer, induced caspase-like activity (Ali et al., 2010). On the other hand, feeding L-NAME (*N*-nitro-L-arginine methyl ester), the inhibitor of NO producing key enzyme NOS, along with *P. falciparum* infected blood reduced the levels of apoptotic ookinetes and ultimately increased the number of developing oocysts (Luckhart et al., 1998). In addition, the reduction of NOS gene expression after silencing the mosquito STAT, produced similar effect on *Plasmodium* as observed above in case of L-NAME treatment (Gupta et al., 2009). Thus, we can conclude that the regulation of *Plasmodium* development inside the mosquito midgut is a multifaceted phenomenon and has been exploited by a number of transmission blocking strategies. For example, the mosquito midgut specific molecules such as alanyl amino peptidase 1 (APN1), carboxypeptidase B (CPB) and a heme peroxidase HPX15 are proposed to design transmission blocking vaccines because inhibition of these molecules suppressed *Plasmodium* development (Lavazec et al., 2007; Atkinson et al., 2015; Kajla et al., 2015b).

### Apoptosis in Ookinete-Invaded Midgut Epithelial Cells is Beneficial for the Mosquito Host

*Plasmodium* ookinetes are motile and cross the midgut epithelium around 20 h after ingestion and targeted by the mosquito immune system. Numerous mosquito immune pathways such as, peptidoglycan recognition proteins (PGRP), scavenger receptors (SCRs), C-type lectins (CTLs), and the genes regulating melanization cascade are reported to induce apoptosis in ookinetes during their traversal to the mosquito midgut epithelium (Michel and Kafatos, 2005). Ookinete invasion-induced expression of NOS and peroxidases mediate the nitration of invaded midgut cells. This nitration process activates mosquito complement TEP1 that mediate ookinete death (Kumar et al., 2004; De Almeida Oliveira et al., 2012; Garver et al., 2013). Ookinete invasion also induces apoptosis in midgut epithelial cells that is revealed by characteristic apoptotic features and commonly observed in *A. gambiae*- *P. berghei*, *A. gambiae*- *P. falciparum*, and *Aedes aegypti*- *P. gallinaceum* combinations. At the last stage of apoptosis, the invaded cells extrude from the epithelial layer into the gut lumen and are replaced by the new cells (Han et al., 2000; Zieler and Dvorak, 2000; Kumar et al., 2004; Gupta et al., 2005). It is important that for a successful invasion the traversing ookinetes must escape unharmed before the invaded cells extrude into the lumen.

The apoptosis of midgut cells is beneficial for the mosquito host because it tends to remove the ‘slow moving ookinetes’ that are entrapped inside the damaged cells. Studies found that in case of *A. stephensi*, the bulged out epithelial cells are found in midgut lumen and *P. falciparum* ookinete are entrapped inside them (Baton and Ranford-Cartwright, 2004, 2005). Our observations also revealed that in the same mosquito midgut cells undergo apoptosis soon after they are invaded by the *P. falciparum* ookinetes (Kumar and Barillas-Mury, 2005). Although the *P. berghei* ookinete invasion induces cell death in mosquito midgut epithelium, however, in this case the ookinetes entrapped inside the dead cells are rarely observed. It may be simply due to the reason that *P. falciparum* infected mosquitoes are maintained at 28°C and *P. berghei* infected mosquitoes at 20°C. This lower temperature maintenance might slow down the midgut cell death processes as reported in case of some mammalian cells (Sakurai et al., 2005).

The invasion of *Anopheles* mosquito midgut by *P. falciparum* and *P. berghei* ookinetes is also different in many ways. In the former case, generally less number of ookinetes successfully completes the invasion and develops into the oocysts. However, in the case of *P. berghei* the rate of ookinete invasion is mostly higher in comparison to the *P. falciparum* infection (Smith et al., 2014). The rapid turnover of mosquito midgut epithelial cells in case of *P. falciparum*, due to their maintenance at 28°C, may be associated with less successful invasion events. In this case, the damaged cells undergo a rapid removal from the epithelial surface and are replaced by the new cells. These dead cells also take away the pathogens that invade them as the part of an intrinsic defense mechanism. The phenomenon of rapid epithelial turnover in reducing the infection by microbial intruders has been observed in other enteric infections. For example, in case of gastrointestinal habitants *Helicobacter pylori* and *Trichuris muris* nematode infections, the gastric epithelial cells undergo apoptosis and expulsion. The increased rate of epithelial turnover makes the mouse resistant against the worm infection (Moss et al., 1996; Kim et al., 1998; Cliffe et al., 2005).

It is of note that the mosquito midgut has few hundred cells; thus, the death in large extend of these cells during ookinete invasion may also be deleterious to the insect host. In other words, the higher density of *Plasmodium* infectious stages in the ingested blood might have deleterious effects on mosquito host. Thus, the balanced regulation of midgut epithelium apoptosis in these mosquitoes is important for their survival after *Plasmodium* infection.

### Ookinete Tends to Suppress Midgut Cell Apoptosis for Its Own Survival

It is clear from the above discussion that to establish infection in the mosquito midgut, ookinetes should escape before they are destroyed or extruded along with the damaged epithelial cell. We believe that in this situation *Plasmodium* must be manipulating either the midgut epithelial immunity or apoptosis in the target cell. Studies carried in *A. gambiae* mosquito revealed that the ookinete protein Pfs47 prevents the activation of caspases and inhibits Jun-N-terminal kinase-mediated activation of apoptosis in invaded mosquito midgut cells (Ramphul et al., 2015). In this study, the wild type parasites regulated broad changes in gene expression profile of the mosquito midgut, however, the Pfs47 mutants failed to do so. Evidences obtained from the above study revealed that the *Plasmodium* death reduced the parasite load in mosquito midgut to support the survival of the vector, completion of sexual life cycle and disease transmission to the vertebrate host. On the other hand, *Plasmodium* mediated interference to the apoptosis of mosquito midgut epithelium also supports its own survival. It manipulates the expression of peroxidase/oxidase system responsible for the nitration of ookinetes, which causes parasite lysis by TEP1 (Ramphul et al., 2015).

### The Death of Oocysts is Mediated by the Mosquito Late Phase Immunity

The ookinete that successfully traverses the midgut are further transformed into the oocyst. The phenomenon of cell apoptosis is not observed in oocysts. However, recent studies reveal that in *A. gambiae* the oocysts encounter a late phase immunity that is mediated through STAT pathway. Silencing of STAT gene in these mosquitoes increased the number of oocysts survival against controls, which was mediated through the reduced expression of NOS, an effector gene of STAT pathway (Gupta et al., 2009). In the same mosquito, an LPS-induced TNFα transcription factor (LITAF)-like 3 (LL3) is also reported to mediate the late phase immunity. LL3 binds to the promoter region of an anti-plasmodial gene termed as serine protease inhibitor 6 (SRPN6) and modulates its expression. Silencing of LL3 gene restricts the differentiation of hemocytes and their responses to parasite infection that results in the increased number of oocysts in the silenced mosquitoes. Thus, LL3 implicates late-phase immunity against *Plasmodium* oocysts through hemocytes (Smith et al., 2012, 2015). These observations might call upon to understand the correlation between the STAT and SRPN 6 pathways in terms of regulating the late phase immunity against *Plasmodium* oocysts.

### Death Process in the Sporozoites

Sporozoites released from the oocyst after several rounds of mitotic divisions although undergo a death process; however, this is a type of non-apoptotic death. They are released in 1000s and 10–20% of them finally invade the salivary glands. Rests of the sporozoites are cleared from the mosquito circulation by hemocytes-mediated phagocytosis (Foley, 1978; Hernández-Martínez et al., 2002; Hillyer et al., 2003, 2007). In salivary glands, *Plasmodium* uptake occurs via a receptor-mediated endocytosis or through a specific ligand interaction. No cytoskeleton rearrangement or apoptosis has been observed in the gland epithelium (Ghosh and Jacobs-Lorena, 2009; Mueller et al., 2010).

*Plasmodium* entry in salivary gland epithelial layer is dissimilar to the midgut epithelium invasion. Salivary glands mount an acute immune response against invading sporozoites. Studies found that some common genes exhibit similar regulation in both epithelia. For example, *Plasmodium* invasion downregulates a fatty acid synthase (AGAP009176) however, GTP-binding nuclear protein, lysosomal thioreductase precursor, and SRPN6 were up-regulated in both the tissues (Rosinski-Chupin et al., 2007). Interestingly, the silencing of *SRPN6* gene in mosquito midgut and salivary gland increased the number of parasites in the respective organ (Abraham et al., 2005; Pinto et al., 2008). This indicates that mosquito innate immunity regulates *Plasmodium* number at different stages of development. This provides an opportunity to manipulate mosquito immunity to control the vectorial competence.

### *Plasmodium* Infection Mediated Apoptosis in Non-target Mosquito Cells

*Plasmodium* does not directly interact with some mosquito cells; however, the infection exhibits an indirect effect on the apoptosis of some other host cells. For example, *P. yoelii nigeriensis* infection in *A. stephensi* resulted in characteristic apoptosis of cells in ovaries or the follicular epithelium and reduced egg production by the gravid females (Hopwood et al., 2001; Ahmed and Hurd, 2006). In addition, the follicular epithelial cell apoptosis is also the major trigger of follicular reabsorption. The follicles showing reabsorption are detected at 12 h that gradually increases to maximum at 24 h post infected blood feeding. Interestingly, at 12 h post feeding the *Plasmodium* development taking place in midgut bolus and at 24 h the ookinete invades the midgut (Han et al., 2000; Kumar et al., 2010). These findings might indicate the advanced detection of *Plasmodium*-induced factors by the mosquito system. In parallel, the anti-plasmodial immunity of mosquito may be responsible for follicular apoptosis through the generation of ROS or RNS. These assumptions are supported by experimental findings where high levels of NO, nitrites/nitrates or ROS are found in *Plasmodium* infected mosquito midgut as well as hemolymph (Luckhart et al., 1998; Crampton and Luckhart, 2001; Kumar et al., 2003). This may be also possible that during the ookinete invasion some bolus bacteria are exposed to the mosquito immune system and the reactive products of innate immunity (ROS and RNS) mediate the degenerative effects in ovaries. The induction of mosquito immunity after lipopolysaccharides (LPS) inoculation into the hemocoel also caused follicular resorption and reduction of fecundity (Ahmed et al., 2002; Hurd, 2003). On the other hand, *Plasmodium* might have developed some mechanisms to induce follicular resorption and channelizing the eggs stored energy for its own development. The understanding of these mechanisms requires further investigations and it can provide a ground to control mosquito fecundity and their population.

## *Plasmodium* Asexual Life Cycle and Cell Apoptosis in Vertebrate Host

*Plasmodium* sporozoites initiate a silent infectious phase called pre-erythrocytic stage inside the liver cells (hepatocytes) and ultimately develop into exo-erythrocytic merozoites (Prudêncio et al., 2011). The sporozoites delivered after mosquito bite in the vertebrate host travel through blood stream and reach liver sinusoids. In the liver sinusoids, sporozoites come to arrest through the interactions of their surface circumsporozoite protein (CSP) with the glycosaminoglycans (GAGs) of stellate cells. According to the gateway hypothesis, the sporozoite glides along the sinusoid wall to locate a Kupffer cell, traverse it and subsequently invade the underlying hepatocyte (Frevert et al., 2005). Recent findings revealed that a heavily glycosylated protein CD68, present exclusively on the surface of the Kupffer cells, acts like a candidate receptor for sporozoite entry. The sporozoites are also reported to remain enclosed within the CD68 endosome that protects them against lysosomal attack within the Kupffer cell. Although the ligand for CD68 receptor is unknown; however, the Kupffer cells of CD68 knockout mouse imposed a barrier for sporozoite invasion (Cha et al., 2015). These findings are important for developing vaccines to block liver stages (LSs) of sporozoites development.

Interestingly, the CD68^+^ Kupffer cells are known to exhibit both phagocytic activity and ROS production capacity (Kinoshita et al., 2010). Thus, the survival of traversing sporozoites must be dependent on the modulation of immune pathways in these macrophages. Studies found that the sporozoite CSP interaction with heparan sulfate proteoglycans (HSPGs) and the low-density lipoprotein receptor-related protein LRP-1 on the surface of these Kupffer cells activates adenylyl cyclase (AC) enzyme. This enzyme, in turn, upregulates cAMP activity and inhibits NADPH oxidase-mediated generation of ROS by the Kupffer cells (Usynin et al., 2007; Kinoshita et al., 2010; Tavares et al., 2013; Cha et al., 2015). These mechanisms create an anti-inflammatory environment that protects sporozoites from the immune attacks in the liver. It is also observed that Kupffer cells exhibit the sign of apoptosis during transformation of sporozoites into early exo-erythrocytic forms (Usynin et al., 2007; Klotz and Frevert, 2008). In conclusion, the sporozoite not only induces desensitization of Kupffer cells to pro-inflammatory stimuli, it also forces their programmed cell death for its own survival.

### *Plasmodium* Manipulates Host Immunity and Apoptosis of Hepatocytes

The intracellular sporozoites are protected from the host immune responses; however, the infected hepatocytes have other mechanisms to eliminate them. Studies have shown that infected hepatocytes undergo apoptosis without external triggers. When sporozoites traverse the hepatocytes, they deform the hepatocyte morphology that results in wounding. This wounding is the trigger for induction of apoptosis in hepatocytes. The apoptosis in infected hepatocytes has been confirmed by previous studies that observed the uptake of fluorescein isothiocyanate (FITC) labeled dextran and propidium iodide by these cells (Mota et al., 2001; Kaushansky et al., 2013). Blocking the apoptosis process in hepatocytes increases LS parasite burden in mice. This suggests that apoptosis of hepatocytes has negative effect on *Plasmodium* progression through hepatocytic stages (Kaushansky et al., 2013). In other words, apoptosis of infected hepatocytes is beneficial for vertebrate host but detrimental for the sporozoites. It is of note that the apoptosis of *Plasmodium* infected hepatocytes exposes the parasitic antigens to initiate protective immune responses in the host (Leiriao et al., 2005b). Therefore, for a successful completion of the LSs, sporozoites must be able to modulate the host immunity and the process of cell death in hepatocytes.

Sporozoites induce the release of hepatocyte growth factor (HGF) from the traversed cells that inhibits tumour necrosis factor (TNF)-mediated apoptosis of hepatocytes. This process helps in successful establishment of the liver infection (Carrolo et al., 2003; Huh et al., 2004; Leiriao et al., 2005a). In addition, sporozoites also inhibit the translocation of PS into the outer leaflet of the cell membrane to block the ‘eat me’ signal displayed by infected hepatocytes to the Kupffer cells. In fact, the swapping of PS into the outer leaflet is driven by the increased cytosolic Ca^2+^. The hepatic stages of parasite sequester this intracellular Ca^2+^ and ultimately inhibit the cellular autophage in hepatocytes (Sturm et al., 2006). A similar mechanism is also displayed by intra-erythrocytic parasites as discussed later.

*Plasmodium* sporozoites are also equipped with other specific mechanisms to manipulate the apoptosis of infected hepatocytes and completing the LSs. Sporozoites mostly invade those hepatocytes that express high levels of EphA2, a transmembrane receptor tyrosine kinase (RTK). EphA2 is generally expressed in the majority of epithelial cells and the interaction of this receptor with its ligand ephrin leads to the contact-dependent cell–cell communication (Park et al., 2013). Interestingly, in hepatocytes the sporozoite remains enclosed inside a parasitophorous vacuole (PV) that is the part of hepatocyte plasma membrane. Recent studies revealed that the interaction of sporozoite surface protein P36 with hepatocyte EphA2 determines the formation of PV. The formation of PV is a viable process to establish a tolerant environment for the replication of sporozoite in the intracellular compartment. In the absence of PV formation, the sporozoites infected hepatocytes suffer extensive cell death and reduce the burden of LSs (Mueller et al., 2005; Kaushansky et al., 2015). Experimental evidences also revealed that the LS infection of *Plasmodium* was largely decreased in EphA2^-/-^ mice in comparison to wild-type mice. This indicated that EphA2 receptor mediated invasion is helpful in the survival of sporozoites and manipulation of apoptosis in the hepatocytes (Kaushansky et al., 2015). Furthermore, studies carried with *P. berghei*-hepatocyte infection stages revealed that the parasite surface protein P36p, the member of P48/45 family of proteins, is responsible for delaying the apoptosis in hepatocytes. It is of note that P36p-deficient *P. berghei* sporozoites are capable of infecting HepG2 cells in culture as well as mouse liver cells *in vivo*. However, the mutant parasites are eliminated faster due to an increased apoptosis in the infected hepatocytes in comparison to the wild type parasite infection. In addition, the mutant parasites also developed the protective immunity *in vivo* (Van Dijk et al., 2005). On the other hand, sporozoite secreted cysteine protease inhibitor also blocks the cysteine proteases of the invaded hepatocytes and suppress their apoptosis (Rennenberg et al., 2010). These findings might conclude that it is a must for the sporozoites to either delay or block the apoptosis of the infected hepatocytes. If the infected liver cell undergoes apoptosis, it will help the antigen presenting cells to display parasite antigens to the acquired immune system. These findings provide newer ways to develop protective immunity against the LSs of *Plasmodium* through manipulation of apoptosis in hepatocytes and demands further exploring this area in details.

### Apoptosis in Erythrocytes Has Dual Effects on Malaria Progression

The enucleated RBCs exhibit programmed cell death known as eryptosis. This phenomenon is similar to apoptosis and characterized by the cell shrinkage, membrane bebbling, and exposure of PS at the cell surface. A major signal of eryptosis is an increased cytosolic Ca^2+^ that involves the cell membrane scrambling in a way similar to the liver cells as discussed before (Föller et al., 2008; Lang et al., 2008). In fact, *Plasmodium* induces oxidative burst that, in turn, activates ion channels in the infected RBC for the uptake of nutrients, Na^+^ and Ca^2+^ ions and excretion of waste product. This Ca^2+^ entry is the major signal for inducing eryptosis (Brand et al., 2003; Tanneur et al., 2006). The phenomenon of eryptosis has been observed in *P. yoelii* 17XL and *P. berghei* ANKA infected RBCs *in vivo* as well as in *P. falciparum* culture. Studies carried in case of *P. yoelii* 17XL revealed that in addition to the parasitized RBCs (pRBCs), non-parasitized RBCs (nRBC) also undergo eryptosis in response to a high parasite load (Eda and Sherman, 2002; Koka et al., 2008; Totino et al., 2010, 2013).

Apoptosis of RBCs has dual role in the advancement of malaria disease. In one way, infected cells undergoing apoptosis are removed by splenic phagocytosis and this process controls the parasite load as well as contributes to the anaemic conditions (Lang et al., 2009; Totino et al., 2010). The death of infected RBCs is the sign of risk for *Plasmodium* development. Thus, to avoid splenic phagocytosis, *Plasmodium*-infected RBCs adhere to the microvascular endothelium via PS and chondroitin sulfate A (CSA) and ends up in more complications such as cerebral malaria, blood–brain barrier (BBB) dysfunction and multiple organ failure (Eda and Sherman, 2002; Setty et al., 2002; Hunt and Grau, 2003; Tripathi et al., 2007; Craig et al., 2012). To overcome the eryptosis-associated side effects, *Plasmodium* tends to delay this process by reducing both the intra-erythrocytic Ca^2+^ levels and the activity of erythrocyte Ca^2+^ pump (Duranton et al., 2003; Huber et al., 2005). Thus, *Plasmodium* should manage the apoptosis of RBCs to balance between the progression of disease and death of the vertebrate host. These mechanisms are important to control erythrocytic stages of *Plasmodium* development. Target specific blockers may be synthesized to manipulate the *Plasmodium*-induced channels in RBC membrane. This will open new opportunities in this field. Several research labs are actively engaged in this area to develop these channel blockers to inhibit the progression of erythrocytic cycle in human malaria (Kang et al., 2005).

## Cell Apoptosis in General is Associated with Severe Complications

Severe complications in malaria are associated with the sequestration of *Plasmodium*-infected red blood cells (pRBCs) in the brain and other organs. Sequestration involves the cytoadherence of pRBCs, which causes over-expression of inflammatory cytokines and target cell apoptosis (Hunt and Grau, 2003; Pino et al., 2003; Wilson et al., 2008). In the severe malaria, vascular integrity is altered as a result of endothelial cell (EC) activation and death, which is caused by the activation of *Plasmodium* apoptosis-linked pathogenicity factors (PALPF), PALPF-2, PALPF-5, and PF11_0521 (N’Dilimabaka et al., 2014). Some findings also suggested that glycosylphosphatidylinositol (GPI) of *P. falciparum* is responsible for the cardiomyocyte apoptosis that reveals malaria associated severity (Wennicke et al., 2008). Acute organ failure (for example, kidney or liver) during complicated malaria is associated with the sequestration and adhesion of pRBCs to the ECs in target organs. In *P. berghei* ANKA infected BALB/c mice high expression of ICAM-1 was found on renal tissues, which is responsible for the interaction of pRBCs with them. This cytoadherence causes change in vascular permeability and recruits inflammatory cells, which contributes to the increased rate of cell apoptosis (Elias et al., 2012). Hence, switching from uncomplicated to severe malaria is possibly caused by the activation of cell apoptosis in different organs. Thus, the target-specific strategies against these major factors that are associated with the disease ‘severity’ might be helpful to control complications during malaria infections.

## Apoptosis in *Plasmodium* is Crucial for Host Survival

*Plasmodium* demonstrates stress responses in vertebrate host and this may result in inducing its own apoptosis. The progression of *Plasmodium* merozoites through intraerythrocytic development includes high fever where temperature of the host body raises up to 41°C. The rise in body temperature is associated with erythrocyte rupture and release of new merozoites. This high temperature can be a natural stimulus for *Plasmodium* to undergo programmed cell death process. *In vitro* studies demonstrated that the DNA fragmentation takes place in *P. falciparum* strain 3D7 after 2 h at 41°C (Oakley et al., 2007). We believe that *Plasmodium* must be equipped by the mechanism that can provide temperature tolerance for its survival.

If the parasite numbers increase beyond an optimal level, it may cause stress and death of the host. In this situation, parasite should adopt a strategy to regulate its own number. The induction of self-apoptosis by the *Plasmodium* may be one of the ways to achieve this goal. *In vitro Plasmodium* culture studies revealed that a growth arrested phenomenon through apoptosis occurs when the parasites undergo a certain percentage of parasitaemia. For example, highly synchronous culture of *P. falciparum* strains Dd2 and 3D7 exhibit cell apoptosis at >11% parasitaemia. Interestingly, these parasites continued their life cycle in the ring stages and failed to progress to the trophozoites and schizonts stages (Mutai and Waitumbi, 2010). This phenomenon of arresting self-development may also reveal a spontaneous judgment by the parasite to protect the host. Host environment also plays an important role in the regulation of *Plasmodium* development. The heme degraded product bilirubin is reported to induce apoptosis in *Plasmodium*. *In vitro* studies demonstrated that the addition of bilirubin to *P. falciparum* culture increased mRNA expression of apoptosis-related genes in the parasites (Kumar et al., 2008). This further suggests that regulating the phenomenon of self-apoptosis in *Plasmodium* might be a good way to control the disease progression in human host. This proposal demands further research in this area.

## Conclusion

The interaction of *Plasmodium* with vertebrate and invertebrate systems is very complex. At different stages of malaria infection, the *Plasmodium* number is regulated by the host immune system or sometime it is a self-directed decision by the parasite. In addition, the program cell death in both the hosts also contributes to this phenomenon; however, *Plasmodium* is capable of suppressing this event to confer its successful development. Thus, the manipulation of apoptosis in *Plasmodium* or host cell might be a promising strategy to control the disease.

## Author Contributions

PK and SS carried the literature survey and contributed to the initial draft. LG and SK wrote the manuscript with input from others. All authors read and approved the manuscript.

## Conflict of Interest Statement

The authors declare that the research was conducted in the absence of any commercial or financial relationships that could be construed as a potential conflict of interest.
